# One-year outcomes of intravitreal brolucizumab injections in patients with polypoidal choroidal vasculopathy

**DOI:** 10.1038/s41598-022-12216-2

**Published:** 2022-05-14

**Authors:** Arisa Ito, Maiko Maruyama-Inoue, Yoko Kitajima, Shoko Ikeda, Tatsuya Inoue, Kazuaki Kadonosono

**Affiliations:** 1 Department of Ophthalmology, Yokohama City University Medical Center, 4-57 Urafune-cho, Minami-ku, Yokohama, Kanagawa 232-0024 Japan; 2 Department of Ophthalmology, Sakae Kyosai Hospital, Kanagawa, Japan

**Keywords:** Medical research, Eye diseases

## Abstract

To evaluate the 1-year visual outcomes and anatomic responses of Japanese patients who received intravitreal brolucizumab (IVBr) injections for polypoidal choroidal vasculopathy (PCV). This was a retrospective study of 17 treatment-naïve eyes with PCV that were treated with IVBr. We evaluated the best-corrected visual acuity (BCVA), central macular thickness (CMT), central choroidal thickness (CCT) and number of injections for 1 year. The eradication of polypoidal lesions was also evaluated using by indocyanine green angiography during the 1-year follow-up. Non-infectious intraocular inflammation developed in two (11.8%) eyes; 15 eyes were assessed at the 1-year follow-up examination. The mean BCVA improved significantly from 0.28 at baseline to 0.13 (*P* < 0.05) at 1 year. The CMT and CCT decreased significantly after 1 year. The mean number of injections was 6.4 ± 0.13. The rate of complete resolution of polypoidal lesions at 1 year was 93.3%. A dry macula was achieved in 13 eyes (86.6%) after the loading phase and in 11 eyes (73.3%) at 1 year. The IVBr injections appeared to be effective for improving both functional and anatomic outcomes in Japanese patients with PCV, with a high regression rate of polypoidal lesions.

## Introduction

Polypoidal choroidal vasculopathy (PCV) was originally described by Yannuzzi et al.^1,2^ as a distinct subtype of wet age-related macular degeneration (AMD). Recently, Spaide et al.^3^ described that PCV is a variant of type 1 macular neovascularization that is more prevalent in Asian individuals. Indocyanine green angiography (ICGA) visualized a branching vascular network and various numbers of aneurysmal dilations at the outer edge of the expanding lesion. In Japan, it has been reported that approximately half of the patients with wet AMD have PCV^4^.

In eyes with PCV, massive hemorrhages and significant loss of vision are evident after a long-term follow-up period^5^. Previous studies have reported that anti-vascular endothelial growth factor (VEGF) agents, such as ranibizumab (Lucentis, Genentech, Inc., South San Francisco, CA) and aflibercept (Eylea, Bayer Health Care, Berlin, Germany) have had been effective in the treatment of PCV, although numerous injections are needed to stabilize patients’ visual acuity (VA)^6–13^. Moreover, aflibercept is more effective than ranibizumab for achieving eradication of polypoidal lesions. However, the eradication rate of polypoidal lesions at 1 year in Japanese patients was not as high as the 13% to 39% reported with ranibizumab^6–9^ and the 39% to 55% reported with aflibercept^10–12^.

Recently, brolucizumab was sanctioned as a new anti-VEGF agent for the treatment of AMD. Brolucizumab is a roughly 26-kDa single-chain antibody fragment^14^. The HAWK and HARRIER studies^15,16^, worldwide phase 3 clinical trials, showed that intravitreal brolucizumab (IVBr) injections administered at every-12-week/every-8-week intervals were effective for improving and stabilizing VA for 96 weeks and were not inferior to the every-8-week dosing interval for intravitreal aflibercept. Moreover, IVBr injections provided better intraretinal, subretinal, and sub-retinal pigment epithelial fluid control than intravitreal aflibercept.

However, the results of IVBr injections for treating PCV in a real-world clinical setting have not been reported. The purpose of this study was to evaluate the 1-year visual outcomes and anatomic responses of Japanese patients with PCV treated with IVBr injections.

## Results

This study included 17 consecutive eyes of 17 Japanese patients with treatment-naïve PCV. Of the 17 patients enrolled in this study, two men developed intraocular inflammation (IOI) and were excluded. Therefore, the 1-year follow-up was completed in 15 eyes of 15 patients (13 men, 2 women; mean age, 77.8 ± 2.7 years; range, 53–90 years).

**Figure 1:**
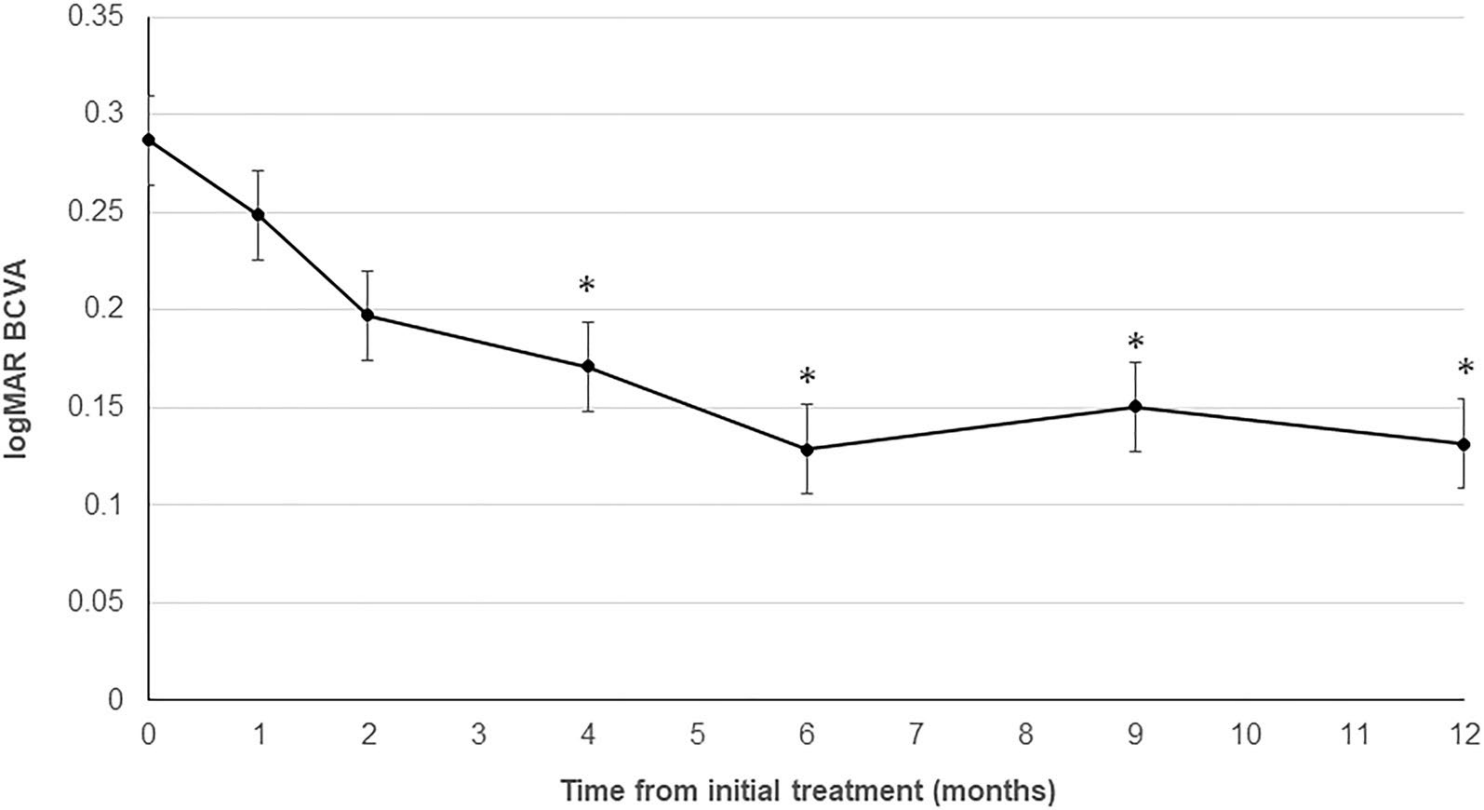
Changes in the best-corrected visual acuity (BCVA) during the 12-month follow-up period. The mean BCVAs at 4, 6, and 12 months improved significantly compared with the preoperative VA (*P < 0.05).

The best-corrected visual acuity (BCVA) improved significantly from 0.28 ± 0.05 (range; -0.079 to 0.69) before treatment to 0.17 ± 0.05 after the loading phase (4 months after starting treatment; *P* < 0.05). The BCVA was maintained at 0.12 ± 0.04 (*P* < 0.05) at 6 months and 0.13 ± 0.06 (*P* < 0.05) at 1 year (Fig. 1).

**Figure 2:**
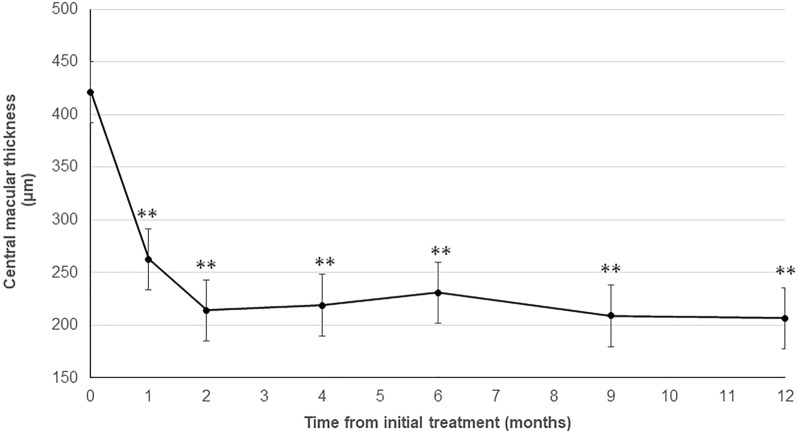
Changes in the central macular thickness (CMT) during the 12-month follow-up period. The mean CMTs at 4, 6, and 12 months decreased significantly compared with baseline (**P < 0.01).

The central macular thickness (CMT) decreased significantly from 421 ± 53 µm before the initial treatment to 219 ± 26 µm at 4 months (*P* < 0.01) and was maintained at 230 ± 22 µm (*P* < 0.01) at 6 months and 206 ± 18 µm (*P* < 0.01) at 1 year (Fig. 2).

**Figure 3:**
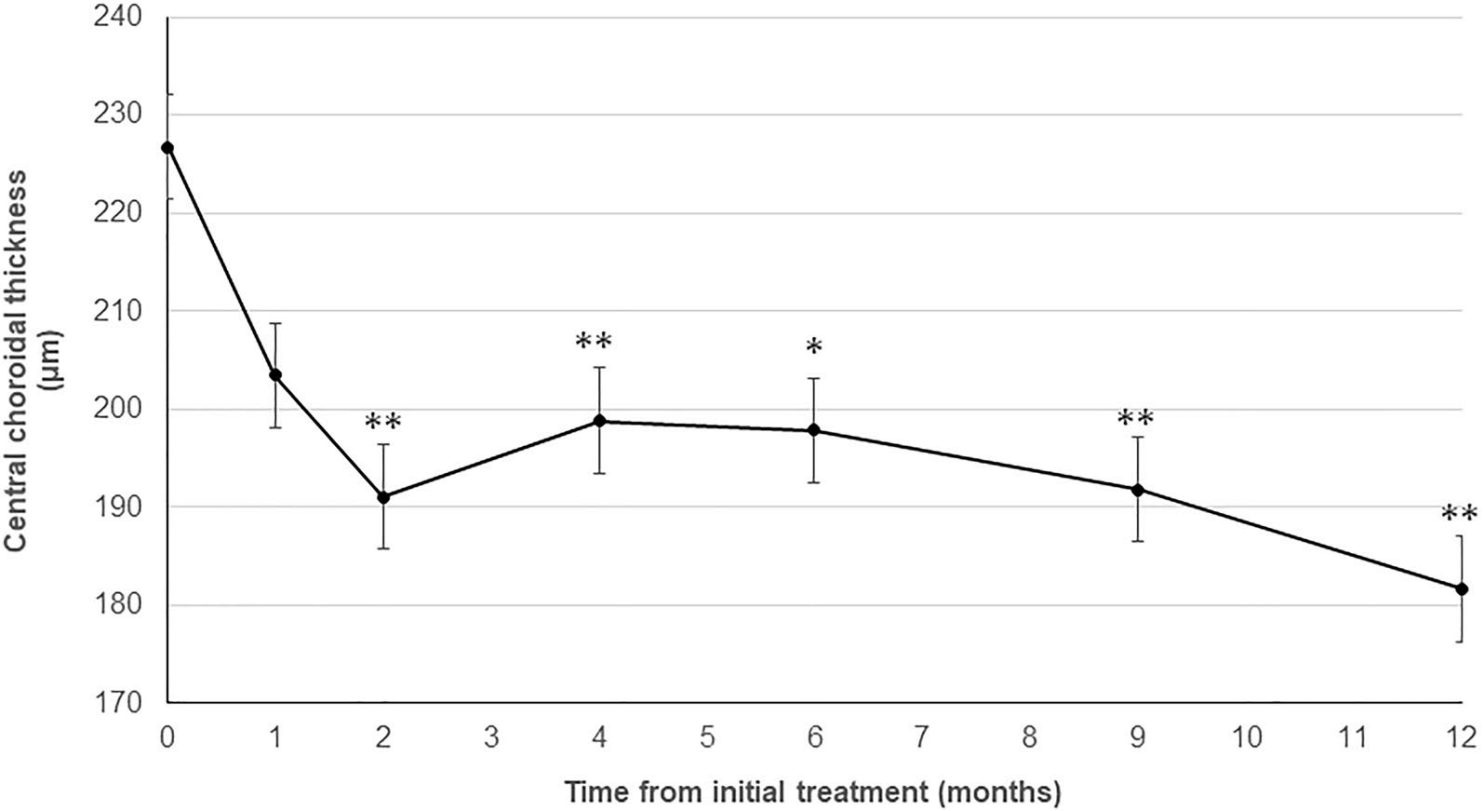
Changes in the central choroidal thickness (CCT) during the 12-month follow-up period. The mean CCTs at 4, 6, and 12 months decreased significantly compared with baseline (*P < 0.05, **P < 0.01).

The central choroidal thickness (CCT) decreased significantly from 226 ± 35 µm before the initial treatment to 198 ± 32 µm at 4 months (*P* < 0.01) and was maintained at 197 ± 31 µm (*P* < 0.05) at 6 months and 181 ± 30 µm (*P* < 0.01) at 1 year (Fig. 3).

During the 1-year follow-up, the mean number of injections administered was 6.38 ± 0.14. After the loading phase, nine patients (60.0%) continued maintenance treatment every 3 months. The treatment interval for four patients (26.6%) was shortened from every 3 months to every 2 months due to the appearance of fluid or new hemorrhage. Two patients (13.3%) continued maintenance treatment every 2 months due to the residual fluid after the loading phase.

**Figure 4:**
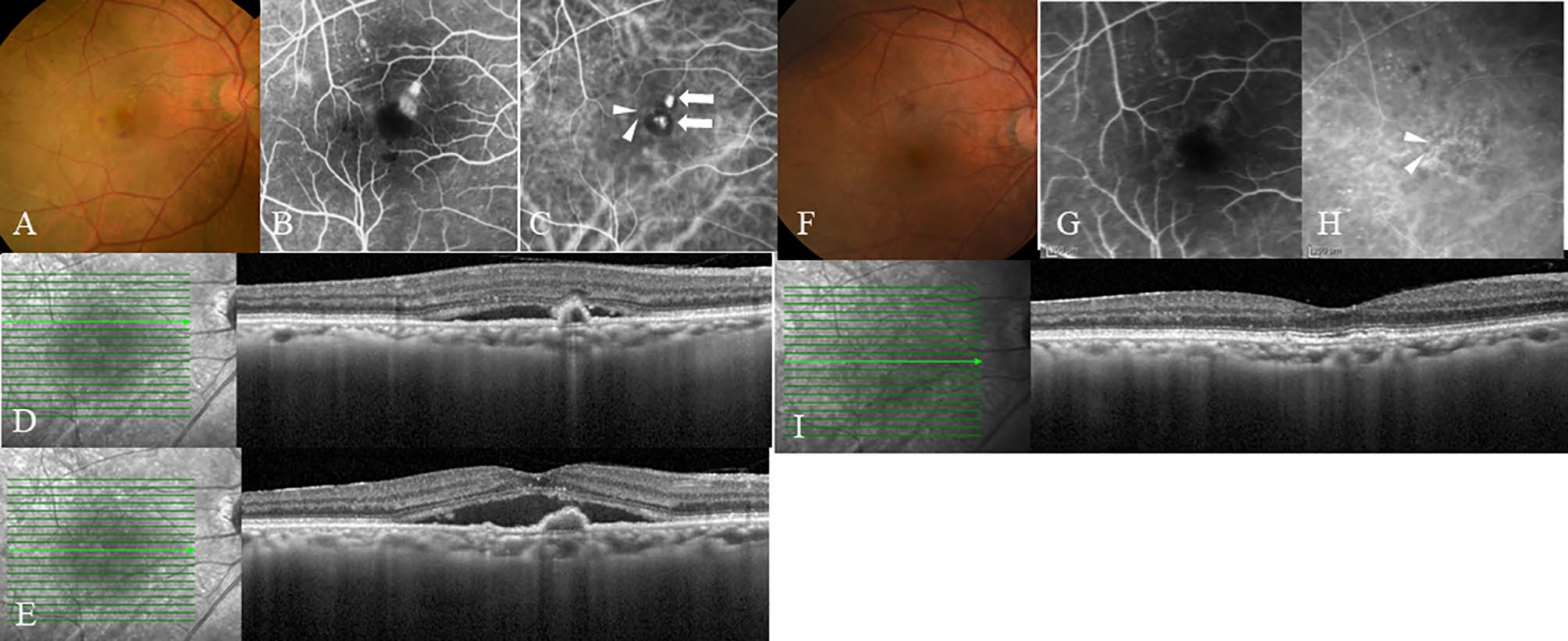
The case of an 86-year-old man who presented with reduced visual acuity in his right eye. (**A**) A color fundus photograph shows reddish-orange polypoidal lesions, including his fovea, submacular hemorrhage, and a large area of subretinal fluid (SRF). (**B**) Fluorescein angiography (FA) demonstrates occult leakage. (**C**) Indocyanine green angiography (ICGA) shows two polypoidal lesions (white arrows) and an abnormal vascular network (arrow heads). (**D**, **E**) An optical coherence tomography (OCT) images obtained at baseline show SRF with polypoidal lesions. (**D**　through the superior polypoidal lesion, **E** through the inferior polypoidal lesion involving the fovea) The visual acuity (VA) was 0.39 logarithm of the minimum angle of resolution (logMAR) in the right eye, and the patient was diagnosed with PCV. The patient received IVBr injections during the loading phase and during the maintenance phase, he was treated every 3 months IVBr injections. The exudative changes did not recur for 1 year. Twelve months after the first injection, the VA improved to 0.045 logMAR. (**F**) A color fundus photograph shows no reddish-orange lesion or hemorrhage at the macula. (**G**) FA shows staining with no leakage. (**H**) ICGA shows complete polyp regression, although an abnormal vascular network (arrow heads) remained. (**I**) OCT shows no polypoidal lesion or SRF. Irregular retinal pigment epithelium elevation was observed where the abnormal vascular network was located.

The proportion of patients with complete regression of polypoidal lesions after 1 year seen on ICGA was 93.3% (14 eyes). A dry macula was achieved in 13 eyes (86.6%) after the loading phase and 11 eyes (73.3%) at 1 year. One eye of four eyes without dry macular at 1 year was the eye without regression of the polypoidal lesion. Figure 4 shows a representative case.

Brolucizumab-related IOI developed in two (11.8%) of 17 eyes. A 72-year-old man had a vitreous opacity after the third injection. His visual acuity reduced from 0.22 logMAR units at baseline to 0.30 logMAR units. 0.1% betamethasone eye drops were started four times a day. A vitreous opacity resolved after two months, therefore he stopped using eye drops. 10 months after IOI was diagnosed, the patient’s visual acuity improved to 0.22 logMAR units, and the fundus examination showed no recurrence of vitreous haze. OCT showed no recurrence of exudation. Another man, 63-year-old man, had vascular sheathing without retinal vascular occlusion after the fourth injection. His visual acuity did not reduce from 0.15 logMAR units at baseline to -0.079 logMAR units. The patients visited the hospital three months after the fourth injection without any symptoms. Therefore, we could not treat the prompt treatment. Four months after vascular sheathing was diagnosed, the patient’s visual acuity was maintained. The fundus examination showed vascular sheathing in the peripheral retina remained, however there was no enlargement of the lesions or the appearance of vascular occlusion. OCT showed recurrence of exudation. Thereafter, the patient received additional intravitreal aflibercept injections. In both cases, the BCVA after 12 months did not deteriorate compared to the baseline BCVA. No other severe adverse events, such as infectious endophthalmitis, rhegmatogenous retinal detachment, cerebral infarction, or myocardial infarction, occurred.

## Discussion

We investigated the 1-year outcomes of IVBr injections in Japanese patients with treatment-naïve PCV and found that the BCVA improved significantly during the follow-up period. The CMT and CCT also decreased significantly after 1 year. The polypoidal lesions regressed completely after 1 year in 93.3% of eyes. To the best of our knowledge, no previous studies have evaluated the regression rate of polypoidal lesions regarding the 1-year outcomes following IVBr injections for patients with PCV.

In a sub-analysis of the HAWK study, Ogura et al.^17^ reported the efficacy of brolucizumab in Japanese patients with PCV. They reported brolucizumab (n = 39) was as good as aflibercept (n = 30) in improving vision. Yamamoto et al.^12^ conducted a multicenter study and reported the 1-year results in 90 treatment-naïve eyes with PCV treated with bimonthly intravitreal aflibercept and reported a mean BCVA improvement from 0.31 to 0.17 logMAR. The mean number of injections during the first year was 7.1. Morimoto et al.^18^ reported the 2-year results in 58 treatment-naïve eyes with PCV with good initial BCVA treated with intravitreal aflibercept using a treat-and-extend (TAE) regimen and found a mean BCVA improvement from 0.27 to 0.12 logMAR during the first year. The mean number of injections during the first year was 7.72. In the current study, the mean BCVA improved from 0.28 to 0.13 logMAR. The number of injections was 6.4. Although a direct comparison between our study and previous results is difficult, our visual outcomes using brolucizumab seem favorable, with fewer injections compared with aflibercept in Japanese patients with PCV.

In the current study, the CCT decreased significantly from 226 to 181 µm (19.9% decrease from baseline) at 1 year. We previously reported the 1-year results in treatment-naïve PCV treated with bimonthly intravitreal aflibercept that had a mean CCT decrease from 265 to 231 µm (12.8% decrease from baseline)^11^. Koizumi et al.^19^ also reported changes in subfoveal choroidal thickness after intravitreal aflibercept injections in 86 patients with PCV at 1 year and reported a mean CCT decrease from 270 to 234 µm (13.3% decrease from baseline). Therefore, the reduction of the choroidal thickness in patients with PCV treated with IVBr injections tended to be greater than that achieved with aflibercept. Kim et al.^20^ reported decreased choroidal thickness was observed after anti-VEGF therapy for PCV. Choi et al.^21^ studied 88 patients with PCV who received anti-VEGF injections and reported that faster chorioretinal atrophy (CRA) growth was related significantly to a decreased choroidal thickness. A larger decrease in choroidal thickness might be a risk factor for long-term development of CRA in patients with PCV.Choroidal thinning might affect the CRA in the longer term and CRA might be related to the BCVA. Therefore, it is necessary to consider whether IVBr injections affect the VA for a longer follow-up period.

In the current study, 60.0% of the patients with PCV completed the 12-week interval injections for 1 year. A dry macula was achieved in 73.3% after 1 year. Ogura et al.^17^ reported the efficacy of brolucizumab in Japanese patients with PCV in a sub-analysis of the HAWK study. The authors reported that the probability of 12-week-interval injections after a loading phase through 48 weeks was 76%, which was similar to our result. The 12-week-interval injections were the longest intervals in the TAE regimen, which is widely used worldwide. The persistence of brolucizumab might derive from its high affinity for VEGF. Its low molecular weight allows more delivery of drug per injection compared with other available anti-VEGFs and offers the potential for more effective tissue penetration and increased duration of action^14^. Therefore, brolucizumab might reduce the treatment burden for patients with PCV.

In the current study, complete regression of polypoidal lesions at 12 months was achieved in 93.3%. Matsumoto et al.^22^ and Fukuda et al.^23^ reported the short-term outcomes of IVBr for PCV. In these two reports, the regression rates of the polypoidal lesions after 3monthly IVBr injections were 78.9% (15eyes of 19eyes) and 78.6% (11 eyes of 14 eyes). There were no previous studies which reports the regression rate of polypoidal lesions regarding the 1-year outcomes. In our previous study, when intraocular aflibercept monotherapy was used to treat PCV, complete regression of polypoidal lesions was achieved in 48.0% with a bimonthly fixed regimen and in 52.9% with an as-needed regimen^11^. In the EVEREST II study^8^, which compared the effectiveness between ranibizumab monotherapy with photodynamic therapy (PDT) combination therapy, the rates at 12 months were 69.3% in the patients treated with PDT/intravitreal ranibizumab therapy and 34.7% in patients treated with intravitreal ranibizumab monotherapy. In another of our previous studies, the regression rates of polypoidal lesions in patients treated with combination therapy with either intravitreal ranibizumab or intravitreal aflibercept were higher (78.2% and 78.9%) than either monotherapy with those drugs^24^. In the current study, the regression rate of polypoidal lesions with IVBr injections was much higher than with either ranibizumab or aflibercept monotherapy or PDT combination therapy, which might be why 73.3% of the patients had a dry macula at 1 year.

However, brolucizumab-related IOI is a major adverse event associated with intravitreal brolucizumab. In the current study, two (11.8%) of 17 eyes had IOI. The frequency of IOI was roughly the same compared with a sub-analysis of HAWK study which Ogura et al. reported (15.4%)^15–17^. Although IVBr injections for PCV are effective, we should be cautious about IOI and treat promptly if brolucizumab-related IOI develops.

This study had some limitations, a major one being the retrospective design. The second limitation was the small sample size. A large-scale randomized study should confirm the current results. Furthermore, the long-term outcomes are unknown. The results of this study must be evaluated with a longer follow-up period.

In conclusion, IVBr injections effectively improved the vision and exudative changes in patients with PCV over a 1-year follow-up, along with higher resolution rate of polypoidal lesions as seen on ICGA. The results of this analysis suggested that brolucizumab could be useful to treat PCV with a lower treatment burden.

## Methods

We retrospectively studied 17 consecutive eyes of 17 Japanese patients aged 50 years or older with previously untreated PCV. All patients received initial treatment using IVBr injections at Yokohama City University Medical Center between June 2020 and February 2021. The institutional review board of the Yokohama City University Medical Center approved this study, which followed the tenets of the Declaration of Helsinki. All patients provided written informed consent before their medical record data were used in research.

All patients received comprehensive ophthalmic examinations, including BCVA, slit-lamp biomicroscopy, optical coherence tomography (OCT) (Spectralis Product Family Version 5.3; Heidelberg Engineering, Heidelberg, Germany), fluorescein angiography (FA) and ICGA (Spectralis Product Family Version 5.3; Heidelberg Engineering, Heidelberg, Germany). The diagnosis of PCV was based on previous reports^4,6^. Inclusion criteria included newly diagnosed PCV with the presence of polypoidal choroidal vascular lesion with branching vascular networks on ICGA. Exclusion criteria were previous history of AMD, history of laser photocoagulation, PDT, intravitreal injection of other anti-VEGF agents or intravitreal steroids. Patients with history of eye diseases such as uveitis, retinal vein occlusion, rhegmatogenous retinal detachment, diabetic retinopathy were also excluded.

During the loading phase, the patients received three monthly IVBr injections. In the maintenance phase, patients received an IVBr injection every 12 weeks unless any fluid or a new hemorrhage was identified, in which case the interval between IVBr injections was shortened to every 8 weeks. The endpoints were the BCVA, CMT, CCT, number of intravitreal injections, regression of polypoidal lesions evaluated by ICGA after 1 year, and complications. These endpoints were evaluated retrospectively 1 year after the initial treatment. CMT and CCT were determined by using a caliper function of B-scan OCT. The CMT was defined as the thickness from the internal limiting membrane to the retinal pigment epithelium at the center of the fovea; the CCT was defined as the thickness between Bruch’s membrane and the inner surface of the choroidal-scleral junction at the fovea. The statistical analysis was performed with Ekuseru-Toukei (Social Survey Research Information, Tokyo, Japan). The BCVA measured on a Landolt chart was converted into the logarithm of the minimum angle resolution (logMAR) for statistical analyses. Wilcoxon signed-rank test was used to compare the changes of BCVA, CMT, and CCT between baseline and other time point. A value of *P* < 0.05 was considered statistically significant.
